# Use of Ivermectin and Chlorine Dioxide for COVID-19 Treatment and Prophylaxis in Peru: A Narrative Review

**DOI:** 10.7759/cureus.31836

**Published:** 2022-11-23

**Authors:** Angie de los Milagros Farfán-Castillo, Rosangela Simone Moreno-Núñez, Fátima Milagros Zárate-Yuyes, Lissett Jeanette Fernández-Rodríguez, Victor Hugo Bardales-Zuta

**Affiliations:** 1 School of Human Medicine, Antenor Orrego Private University, Trujillo, PER

**Keywords:** peru, alternative medicine, covid-19, chlorine dioxide, ivermectin

## Abstract

The spread of severe acute respiratory syndrome coronavirus 2 (SARS-CoV-2), the virus that causes COVID-19, created a rapidly unfolding health crisis, especially in the initial phases of the pandemic. In the early stages of the pandemic, various strategies were proposed for COVID-19 prophylaxis and treatment with very little scientific evidence available. Among these proposed treatments were ivermectin and chlorine dioxide, which were both used widely in Peru for both disease prevention and treatment without considering their problematic side effects. For instance, ivermectin was part of an approved therapeutic scheme based on in vitro data, although its efficacy in humans was not demonstrated. In addition, chlorine dioxide was never shown to be effective but causes threatening side effects. In this article, we discuss current information regarding chlorine dioxide and ivermectin in the context of the COVID-19 pandemic, with a focus on experiences in Peru.

## Introduction and background

The COVID-19 pandemic highlighted deficiencies in healthcare systems worldwide. Peru was no exception: the precarious state of hospitals and lack of life-saving equipment and supplies such as medical oxygen and ventilators resulted in many preventable deaths [1]. Further compounding problems, there was very little scientific literature on which to base the treatment recommendations, especially at the beginning of the pandemic. In Peru and elsewhere, this lack of clear scientific evidence fostered disagreements about which intervention(s) and medicine(s) were safe and effective for COVID-19 treatment and prophylaxis.

As more data became available through research and clinical experience, treatment recommendations were updated frequently: seven modifications were made between March 30, 2020, and June 9, 2020, by the Peruvian Ministry of Health, a period of less than three months [2]. For example, one resolution, approved on May 8, 2020 [3], recommended hydroxychloroquine and ivermectin for mild cases and hydroxychloroquine alone or with azithromycin or chloroquine phosphate and ivermectin for moderate to severe cases.

The frequent changes of recommendations combined with waves of infection and death generated fear and distrust in the medical establishment among the general Peruvian population. These concerns, combined with the spreading of misinformation on social media and profiteering, fostered the promotion and use of "treatments" without scientific evidence, such as chlorine dioxide and ivermectin, usually in an inadequate or excessive manner [4,5].

Here, we compile available and recent information regarding COVID-19 treatment and prevention with ivermectin and chlorine dioxide with a focus on experiences in Peru and references written in Spanish that may not be easily accessible to an English-speaking audience. We evaluated over 500 search results and narrowed this to include only 35 documents, published between January 2020 and June 2022, that met the quality acceptance criteria to be included in this review.

## Review

Ivermectin background and pharmacology

Ivermectin is a pharmaceutical approved for use in both humans and animals. First commercialized in 1987 by Merck Laboratories, the drug was initially used in the African Onchocerciasis Control Program [6]. Ivermectin is a highly lipophilic pharmaceutical (c log P = 5.83) which consists of two very closely related chemicals: 22,23-dihydroavermectin-B1a and to a lesser extent 22,23-dihydroavermectin-B1b [7,8]. It can be administered orally, subcutaneously, topically, or intramuscularly, but only oral administration has been approved for humans [8]. It reaches its highest plasma concentration three to 10 hours after administration, distributes widely throughout the body, and is most persistent in fatty tissues [8]. In the blood, 93.2% binds to plasma proteins [6]. Ivermectin is an extremely potent antiparasitic drug with an optimal oral dose of 150 µg/kg; it is frequently administered in 12 mg doses. Also, it can be safely combined with other antiparasitic drugs and antibiotics. The drug works by blocking glutamate-dependent and γ-aminobutyric acid-dependent chloride channels, which are present in the parasite nervous system, but absent in the mammalian host, thus killing the parasite without harming the host [6,8]. Additionally, this drug has in vitro activity against RNA viruses, such as Chikungunya, influenza A, and Zika. However, its minimum effective dose for viruses is greater than the dose needed to eradicate its original multicellular targets [6-10]. It may also have immunomodulatory effects [9]. The drug is processed by liver CyP450, with significant recycling, and its metabolites are ultimately excreted in the feces [8].

Chlorine dioxide background and pharmacology

Chlorine dioxide is a reactive greenish-yellow gas often obtained by reacting sodium chlorite and/or chlorate with acids and reducing agents. This chemical is a strong and effective water-soluble oxidizer [5,11]. These properties make it ideal for use as a biocide and bleaching agent. It acts by oxidizing and disrupting cellular and viral components such as membranes and proteins [5]. Although it oxidizes a wide range of biological molecules, eukaryotic cells seem to be less susceptible to this damage [12]. It is mostly encountered by the general population at low concentrations as a disinfectant in municipal water, where its concentration is controlled, and as an antiseptic in hospitals [5,13,14]. Additionally, it has been evaluated as a food and dental disinfectant and mouthwash with mixed results [11,12]. When ingested, it is rapidly absorbed through the digestive tract, reaching peak concentration about one hour after dosing. It is slowly absorbed through the skin and can cause severe lung damage if inhaled at high concentration [5,15]. It is metabolized into chlorite, chlorate, and chloride ions and eliminated in the urine and feces [5,15].

Antiviral effect and uses of ivermectin and chlorine dioxide in the context of the COVID-19 pandemic

The use of ivermectin during the COVID-19 pandemic was first suggested in the research article "The FDA-approved drug ivermectin inhibits the replication of SARS-CoV-2 in vitro" [16]. Additional investigations were carried out on the cellular proteins that interact with severe acute respiratory syndrome coronavirus 2 (SARS-CoV-2), finding that importin α/β1-heterodimer (IMPα/β1) transports viral nucleocapsid and ORF6 proteins to the nucleus where it diminishes the signal transducer and activator of transcription 1 (STAT1)-mediated host response to viral infection [6,17-20]. Ivermectin was shown to interfere with IMPα/β1 formation, thus leaving cellular viral defenses intact. It is worth noting, however, that these effects were demonstrated in vitro [6,16]; it remains to be proven in large studies that ivermectin can effectively treat COVID-19.

Chlorine dioxide was already being marketed in the United States and Europe as a disinfectant and “cure-all” before the coronavirus pandemic. One common product produces chlorine dioxide by combining sodium chlorite and citric acid immediately before use. Since these products were already on the market, they were quickly promoted as COVID-19 prophylactics and treatments [13]. However, the usefulness of this chemical has been rejected by several important organizations such as the Pan American Health Organization and the Peruvian National Institute of Health because there is no available published scientific evidence that has supported the use of chlorine dioxide for COVID-19 prevention or treatment, whether administered orally or by inhalation [14,21].

What are the adverse side effects of ivermectin and chlorine dioxide?

The reported adverse effects of excessive use of ivermectin include nausea [20], acne [20,22], dizziness [22], abdominal pain [23], eosinophilia [23,24], neurotoxicity symptoms [23], diarrhea [24], and blurred vision [23,24]. High doses of ivermectin cross the blood-brain barrier, altering GABAergic communication, and consequently causing alterations in the central nervous system. In addition, cases have been reported where repeated dosing above the therapeutic recommendations caused side effects such as tachycardia, vomiting, changes in the electrocardiogram, drowsiness, and ataxia and significant variations in blood pressure and mydriasis [20].

The ingestion of chlorine dioxide can produce serious adverse effects, including acute liver failure, hemolytic anemia, respiratory failure due to methemoglobinemia, hydroelectrolytic imbalance (hypotension), prolongation of the QT interval that causes arrhythmias, eye irritation, and severe diarrhea and vomiting [25,26]. Other adverse effects include nausea [25,26], esophagitis [5], bronchospasm [26], pruritus [26], and chemical pneumonitis [25].

Chlorine dioxide and ivermectin treatment for COVID-19

The present research on therapeutic and/or prophylactic ivermectin and chlorine dioxide on SARS-CoV-2 infection and COVID-19 mortality is compiled in Table 1.

**Table 1:** Summary of current published literature on the use of ivermectin and chlorine dioxide for COVID-19 prophylaxis and treatment. SARS-CoV-2: severe acute respiratory syndrome coronavirus 2.

Drug	Use case	Observation	Evidence quality
Ivermectin	Therapeutic	Mortality rate reduction [27]	Based on 280 consecutive patients in four hospitals
Ivermectin	Prophylactic	73% reduction in SARS-CoV-2 [28]	Matched case-control study, 186 pairs
Ivermectin	Therapeutic	All-cause mortality rate reduction [29]	No definitive evidence supporting the use
Ivermectin	Therapeutic	A five-day course of drug eliminates the virus faster than a placebo (p = 0.005) [30]	Randomized clinical control study is necessary to validate the result
Ivermectin	Therapeutic and prophylactic	Treatment with the drug did not reduce the incidence of hospitalization for COVID-19 [31]	No definitive evidence supporting the use
Ivermectin	Therapeutic	Drug has good safety profile but did not reduce the need for supplemental oxygen, ICU admission, invasive ventilation, or death among patients hospitalized for COVID-19 [32]	No definitive evidence supporting the safety and efficacy
Ivermectin	Therapeutic	Ivermectin may provide increased clinical recovery, improved prognostic laboratory parameters, and decreased mortality rates [33]	This study needs to be repeated with a larger cohort
Chlorine dioxide	Therapeutic and prophylactic	There is no published scientific literature supporting use to decrease COVID-19 mortality [26]	No scientific evidence supporting the use
Chlorine dioxide	Therapeutic and prophylactic	Use causes serious health problems [34]	No scientific evidence supporting the use
Chlorine dioxide	Therapeutic	No published study that found supports the therapeutic use [13]	No scientific evidence that found supports efficacy and safety
Chlorine dioxide	Therapeutic	No published study that found supports the use [25]	No scientific evidence that found supports the therapeutic use

FDA opinion on ivermectin and chlorine dioxide use

It is possible that ivermectin plays a role in inhibiting viral replication, which makes it a candidate for treating different viral diseases [6,22]. These effects have been demonstrated in vitro, making it necessary to complete large clinical studies to determine whether these effects translate to measurable clinical outcomes. In April 2020, the FDA published a letter to stakeholders that recognizes the results of in vitro studies testing ivermectin activity against SARS-CoV-2 which are common in the early stages of drug development [35]. The letter recommends that ivermectin be used in an FDA-approved manner, under the supervision of a doctor. Additionally, it cautions against self-medication and the use of forms of ivermectin intended for veterinary use. This is because sufficient clinical evidence that ivermectin is a safe and effective treatment or prophylactic against SARS-CoV-2 is lacking. Also, its uncontrolled use can result in serious side effects.

Although the safety and efficacy of chlorine dioxide used as a medicine are contradicted in the scientific literature (Table 1), some people insist on using it as a “cure-all” against such diverse diseases as the flu, HIV, autism, cancer, and COVID-19. Because of this, the FDA published an update on April 8, 2020, warning against marketers of chlorine dioxide making unsupported claims for their product. It further states that there is no scientific evidence that chlorine dioxide is effective against SARS-CoV-2 and that its use can cause serious side effects, including death, especially in children. Using chlorine dioxide may also delay proper diagnosis and treatment [36].

Why are these drugs being used in Peru for COVID-19?

The Peruvian Health Ministry (Ministerio de Salud (MINSA)), in agreement with other health organizations, stated on May 8, 2020, that to date there is no scientific evidence to recommend specific treatments for COVID-19 patients. However, the document includes various treatment suggestions, including 200 µg ivermectin per kg in a single oral dose for moderate cases, and two daily doses for severe cases. Similarly, they recommend that health personnel monitor for adverse reactions since the use of ivermectin in humans for COVID-19 treatment has not been approved by the FDA [3].

The General Directorate of Medicines, Supplies, and Drugs of Peru (DIGEMID) warns of a risk of potentially serious harm to those who consume chlorine dioxide for therapeutic or prophylactic use. It goes further to state that the promotion and marketing of chlorine dioxide-containing “medicines” [37] is an illegal act. Likewise, the National Institute for the Protection of Competition and Intellectual Property (INDECOPI) pointed out that this product “could be harmful to human health, since it does not have scientific support, authorization, or Sanitary Registry” [38].

## Conclusions

Although the development of SARS-CoV-2 vaccines and drugs has likely saved the lives of millions, the search for additional efficacious and safe antiviral drugs should continue, as COVID-19 will most certainly not be the last pandemic. In this quest, ivermectin was an early front-runner in treatment, but new antivirals, such as Paxlovid, are more potent inhibitors of viral replication. At present, Paxlovid is not yet approved for use in Peru, thus decreasing COVID-19 treatment options. It is recommended that the appropriate authorities approve this drug to improve the prognosis of Peruvian COVID-19 patients.

Ivermectin use has been shown in multiple studies to associate with lower mortality during COVID-19 treatment. However, these studies are limited and include a small sample size resulting in a low level of scientific certainty, which is insufficient to make general recommendations for treatment. Ideally, randomized controlled trials with a sufficient study population would be better to obtain consistent conclusions about whether this drug can improve prognosis.

We were unable to find any scientific evidence for the use of chlorine dioxide for SARS-CoV-2 prophylaxis or infection. Its consumption puts the population at risk for serious and often life-threatening adverse reactions, especially when consumed excessively. Chlorine dioxide was never seriously considered a treatment for SARS-CoV-2 infection; instead, it is a case study in the spread of pseudoscientific information in the informal media. Medical professionals should do what they can to dispel this myth and prevent others from becoming prevalent as this and other pandemics unfold. Such actions can include patient education, countering misinformation on social media, and supporting government regulations and funding to promote scientifically based treatments.
